# Improved retroviral suicide gene transfer in colon cancer cell lines after cell synchronization with methotrexate

**DOI:** 10.1186/1756-9966-30-92

**Published:** 2011-10-04

**Authors:** Laetitia Finzi, Aurore Kraemer, Claude Capron, Severine Noullet, Diane Goere, Christophe Penna, Bernard Nordlinger, Josette Legagneux, Jean-Fançois Emile, Robert Malafosse

**Affiliations:** 1 Research center, division of Digestive and Oncologic Surgery, Ambroise Pare Hospital and University of Versailles- Saint-Quentin, Boulogne, France; 2 EA 4340, Ambroise Pare Hospital, Boulogne and University of Versailles-Saint-Quentin, France; 3 Immunology laboratory, Ambroise Pare Hospital and University of Versailles- Saint-Quentin, Boulogne, France; 4 Ecole de Chirurgie, Assistance Publique-Hôpitaux de Paris, Paris, France

## Abstract

**Background:**

Cancer gene therapy by retroviral vectors is mainly limited by the level of transduction. Retroviral gene transfer requires target cell division. Cell synchronization, obtained by drugs inducing a reversible inhibition of DNA synthesis, could therefore be proposed to precondition target cells to retroviral gene transfer. We tested whether drug-mediated cell synchronization could enhance the transfer efficiency of a retroviral-mediated gene encoding herpes simplex virus thymidine kinase (HSV-*tk*) in two colon cancer cell lines, DHDK12 and HT29.

**Methods:**

Synchronization was induced by methotrexate (MTX), aracytin (ara-C) or aphidicolin. Gene transfer efficiency was assessed by the level of HSV-TK expression. Transduced cells were driven by ganciclovir (GCV) towards apoptosis that was assessed using annexin V labeling by quantitative flow cytometry.

**Results:**

DHDK12 and HT29 cells were synchronized in S phase with MTX but not ara-C or aphidicolin. In synchronized DHDK12 and HT29 cells, the HSV-TK transduction rates were 2 and 1.5-fold higher than those obtained in control cells, respectively. Furthermore, the rate of apoptosis was increased two-fold in MTX-treated DHDK12 cells after treatment with GCV.

**Conclusions:**

Our findings indicate that MTX-mediated synchronization of target cells allowed a significant improvement of retroviral HSV-*tk *gene transfer, resulting in an increased cell apoptosis in response to GCV. Pharmacological control of cell cycle may thus be a useful strategy to optimize the efficiency of retroviral-mediated cancer gene therapy.

## Background

Cancer gene therapy by suicide gene transfer remains an alternative approach to increase selectivity in cancer treatment [1]. The enzyme prodrug strategy, involving transfer of the suicide gene, *i.e. *HSV-*tk*, to tumor cells followed by ganciclovir (GCV) treatment, is the most widely used [2-5]. HSV-TK phosphorylates GCV to its monophosphate form that is then converted by cellular kinases into GCV triphosphate, which causes DNA chain termination and cell death [6]. *In vivo*, this strategy involves both a direct cytotoxic effect and a bystander effect [7]. The bystander effect confers cytotoxicity to the neighboring nontransduced cells [8], and a distant anti-tumor immune response. These aforementioned ways for killing tumors are related to the quantitative efficiency of gene transfer [9,10]. However, one of the major obstacles to successful cancer gene therapy is the inadequate transduction of the target cells [11]. *In vivo*, several studies have shown that the number of cells transduced by retroviral vectors constitutes less than 10% of the target cell population [12,13].

The transduction efficiency of defective murine-derived retroviral vectors requires target cells to be in division because integration of the great size viral DNA-protein complex needs the metaphasic breakdown of the nuclear membrane. Integration of the transgene thus depends on the phase of the cycle where the target cells are [14-16]. Consistently, the relationship between cell cycle and retroviral transduction has previously been shown [15,17,18]. The gene transfer efficiency was lower in cultured cells enriched in G0-G1 phase than that in similar cell populations enriched in S, G2 and M phases [18]. The accumulation of cells blocked in a determined cell cycle phase which is the definition of synchronization, could thus improve the efficiency of gene transfer and finally the effectiveness of viral transduction. Consistently, cells need to be synchronized in S phase due to the intracellular half-life of murine retroviruses. Synchronization of cells in S phase can be obtained *in vitro *by serum starvation or by drugs inducing a reversible DNA synthesis inhibition. Methotrexate (MTX), aphidicolin or aracytin (ara-C) have been used to synchronize several cell lines in S phase. The effect of these drugs is reversible in respect with the micromolar concentrations used [19-22]. Although synchronization has been used for improving the efficacy of chemotherapy [23,24], the effect of synchronization on the efficiency of retroviral gene transfer has never been evaluated in colon cancer cells. The aim of this study was to evaluate whether transduction efficiency may be increased by the synchronization of target cells before retroviral gene transfer.

## Methods

### Cell culture

We used two colon cancer cell lines: the human HT29 and the murine DHDK12 pro-b (Pr. Martin, Dijon; France) cell lines. Cell lines were cultured in DMEM medium containing 10% calf serum/penicillin (50 units/ml)/streptomycin (50 μg/ml) at 37°C in 5% CO_2_. We used retroviral vectors carrying *Escherichia-coli *β-galactosidase (β-*gal*) [25] and herpes simplex thymidine kinase (HSV-*tk*) genes associated with pac and neoR gene respectively as positive selectable marker genes. Amphotropic packaging cells were generated from the human embryonic kidney cell line 293. The packaging cells stably express Friend Murine Leukemia Virus (F-MuLV) strain FB29 gag/pol genes and an amphotropic envelop gene derived from pPAM3 (A.D Miller Seattle, WA, USA). Packaging cells were transfected with plasmids pTG 5391 (FB29 LTR-lacZ-SV40-Puro-LTR, clone E17-12 -TG 5391) or pTG 9344 (FB29 LTR-PGK-TK-IRES-Neo -LTR clone E 17-21 pTG 9344) to isolate the retroviral producer clone E17-12 -TG 5391 and E 17-21 TG 9344 (Transgene S.A., Strasbourg, France). The retroviral producer clone were cultured in DMEM supplemented with 4.5 g/L of glucose, 1% non-essential amino acids, 40 μg/ml gentamycin (Sigma) and 10% calf serum. Culture supernatant was harvested, filtered through a 0.45 μm nitrocellulose filter (Sartorius, Goettingen, Germany) and used in the presence of polybrene (Sigma) at 8 μg/ml final concentration. NIH 3T3 fibroblasts were cultured in DMEM supplemented with 40 μg/ml gentamycin and 10% heat inactivated NBBS (GIBCO/BRL). Retroviral titration was determined by infecting NIH 3T3 fibroblasts with serial dilutions of the culture medium and staining respectively for β-galactosidase activity with X-gal protocol [26] or for HSV-TK expression using monoclonal antibody anti-HSV-TK as described below. All point titrations were performed four times. The titer of viral preparation was 4.9 (± 1.2) × 10^6 ^focus-forming units (FFU/ml) for TG 9344 and 1.7 (± 0.9) × 10^7 ^FFU/ml for TG 5391. The absence of competent replication helper retrovirus was checked by NIH 3T3 mobilization assay

### Treatment of cells with MTX, ara-c or aphidicolin

DHDK12 and HT29 cells were plated into 12 well plates at 5.10^5 ^cells/well and treated with 0.08 μM methotrexate (Wyeth-Lederle, Puteaux, France) or 0.075 μM 1-β-D-arabinofuranosyl (Cytarabin-Pharmacia-Upjohn) or 25 μM aphidicolin (Sigma) for 24 hr. The concentrations of the drugs used in our study were chosen according to previously published studies [19,21,22]. Furthermore, we determined the IC50 of these drugs by a growth curve analysis. All concentrations used in our study were lower than the calculated IC50 (Table 1). After treatment, the drug-containing medium was removed; the cells were washed twice with phosphate-buffered saline (PBS) and fresh medium was provided. Every 2 to 6 hr during 72 hr, cell cycle distribution were obtained by flow cytometric determination of the DNA content of propidium-iodide (PI)-stained cells as described previously [27]. The cells were analyzed on a cytofluorometer EPICS XL-MCL (Coulter Beckman, Miami, USA) with an argon laser emitting at a wavelength of 488 nm. The analysis of fluorescence was carried out starting from an acquisition window determined by a two dimensional histogram representing the structure of the cells scaled to their size. This acquisition window was then used to produce a histogram representing the number of PI positive cells sorted by intensity of fluorescence, expressed in logarithmic curve mode.

**Table 1:** IC_50 _of Methotrexate, Ara-C and Aphidicolin in DHDK12 and HT29 cell lines

IC _50_	Methotrexate	Ara-C	Aphidicolin
**DHDK12 cells**	0.16 μM	40 μM	30 μM

**HT29 cells**	0.1 μM	60 μM	30 μM

### Gene transfer into synchronized cells

DHDK12 and HT29 cells were transduced with the reporter gene β-*gal*. After removal of drug-containing medium, samples were taken every 8 hr during 72 hr. For each time, cells were infected with 1 ml of 0.45 μm filtered TG 5391 packaging cells supernatant in the presence of 8 μg/ml of polybrene.

Then, HSV-*tk *gene was used during optimal period determined with the reporter gene for each cell line. During this period, cells were infected with 1 ml of 0.45 μm filtered TG 9344 packaging cells supernatant in the presence of 8 μg/ml of polybrene at various time points after MTX removal.

For each time point, appropriate controls were performed. Transgene expression was determined 48 hr after transduction.

### Transgene expression assay

For detection of β-galactosidase activity, cells transduced by TG 5391 were fixed for 15 min at 37°C with 0.5% of glutaraldehyde, then washed two times with PBS and stained with X-gal for cytochemical analysis, as previously described. The quantitative detection of β-gal expression was performed with the fluorescein-di-β-D-galactopyranoside (FDG) (Sigma) by flow cytometry [28]. Cells were harvested (trypsin-EDTA), washed and resuspended at a concentration of 5.10^5^/ml in 25 μl of PBS containing 2% fetal calf serum, at 37°C for 10 min. The β-galactosidase activity was obtained by cell incubation in 25 μl of 2 mM FDG solution for one min at 37°C, then for one hour at 0°C, in 1 ml of PBS. The fluorescence was analyzed by flow cytometry. Non-transduced cells formed the control group.

For HSV-TK expression analysis, cells transduced by TG 9344, cultured on slides (Labtek II-Nunc), were fixed for 15 min at 4°C with 4% paraformaldehyde and incubated with PBS containing 0.2% serum bovine albumin (SAB) and 0.1% saponin for 5 min. Cells were incubated with anti-HSV-TK mouse monoclonal antibody 4C8 (W. Summers, Yale University, USA) 1/50, for 30 min at room temperature. After washing in PBS, cells were incubated for 10 min in a secondary antibody solution of goat anti-mouse coupled to biotin (LSAB 2 System Peroxydase, Dako). Cells were washed in PBS and incubated 10 min with streptavidin-peroxydase. The revelation was achieved by incubation for 5 min with 3-3' diaminobenzidine (DAB) leading to cytoplasmic brown precipitates. Cells were counterstained with hematoxylin.

For flow cytometry analysis, cells were harvested, washed in PBS and fixed with 4% paraformaldehyde for 15 min at 4°C in PBS. Cells were washed in incubation buffer (0.2% SAB, 0.1% saponin in PBS containing 0.2% of sodium azide) then incubated in 200 μl of anti-HSV-TK monoclonal antibody 4C8, diluted to 1/50 in incubation buffer for 30 min at room temperature. Cells were washed three times with PBS. The pellet was resuspended 30 min at room temperature, in 200 μl of goat anti-mouse antibody coupled to FITC, diluted to 1/100 in incubation buffer. Cells were washed and resuspended in 1 ml of PBS for flow cytometry analysis.

### Measurement of ganciclovir-induced cytotoxicity in synchronized cells

Flow cytometry was carried out on synchronized cell, transduced with TG 9344 at different periods, after 72 hr of 20 μM GCV treatment to quantitate cell apoptosis. Apoptosis was determinate by staining cells with annexin V-FITC and propidium-iodide (PI) labeling, because annexin V can identify the externalization of phosphatidylserine during the apoptotic progression and therefore detect early apoptotic cells [29]. Cells were transduced with TG 9344 vector, on 12-well plates and treated after 24 hr by 20 μM GCV. Control cells were no transduced or untreated. After 72 hr of treatment, cells were harvested, and washed twice in PBS. The pellet was resuspended in 1 ml of 100 mM HEPES/NaOH, pH 7.5. Then 500 μl of the cell suspension were incubated in presence of 2 μg/ml annexin V-FITC, and 10 μl of PI (100 μg/ml) for 10 min. Samples were immediately analyzed by flow cytometry on a bi-parametric histogram giving the level of annexin V-FITC and PI fluorescence.

Apoptosis was assessed by DNA fragmentation assay. Samples of 5.10^5 ^pTG 9344 transduced cells with or without synchronization were treated 96 hr with 20 μM GCV. Cells then were centrifuged at 800 g for 5 min at 4°C. The pellet was resuspended in 20 μl of lysis buffer (EDTA 20 mM, Tris 100 mM, SDS 0,8%, pH 8). Then 10 μl of 500 UI/ml RNAse (Sigma) were added for 60 min at 37°C. The mix was incubated 90 min at 50°C with 10 μl of 20 mg/ml proteinase K. Migration was achieved on 1.8% agarose gel containing 0.5 μg/ml ethidium bromide at 35 V during 4 hr. MSP I digested PBR 322 was used as a size marker. Non-transduced cells treated with MTX or GCV constituted control groups.

### Statistical analysis

Comparisons were made using the Student's t test. P < .05 was considered as significant.

## Results

### Altered progression in the cell cycle by methotrexate, ara-C or aphidicolin

We first assessed the effect of drugs on DHDK12 and HT29 cell cycles to delineate the time for which a maximum of cells were in S phase after drug removal.

The effects of the three drugs, i.e. MTX, ara-C and aphidicolin, on the cell cycle were preliminary assessed in DHDK12 cells. After a 24 hr treatment with MTX, ara-C or aphidicolin, cells were analyzed between 0 and 72 hr after drug removal for DNA content by flow cytometry.

**Figure 1:**
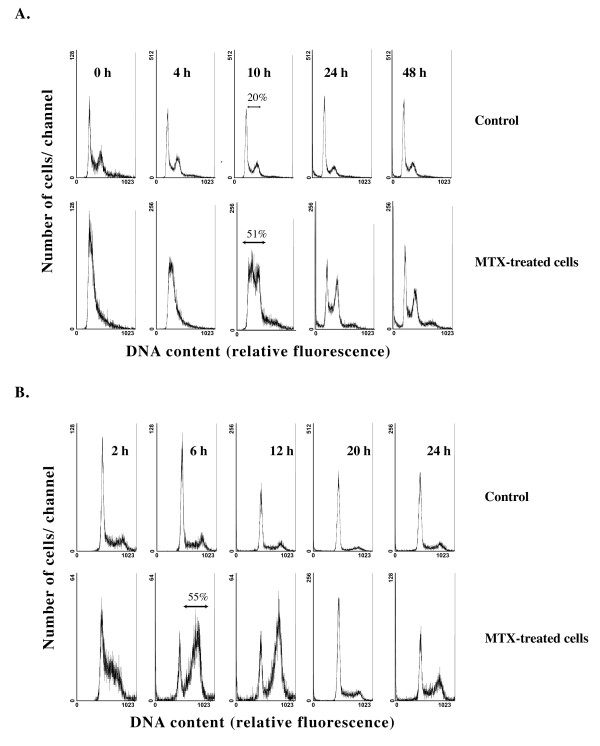
**Distribution in cell cycle-phase after MTX treatment**. Cell cycle phases of DHDK12 cells (**A**) and HT29 cells (**B**) were obtained by uniparametric flow cytometry analysis of DNA content (propidium iodide red-fluorescence intensity in fluorescence units) at various time after MTX removal. On the ordinate is shown the number of cells corresponding to the fluorescence units.

In the DHDK12 cell line, 20% of cells were in S phase in the absence of drug and this rate was constant over time (Figure 1A). When DHDK12 cells were treated with ara-C or aphidicolin, 25% and 35% of cells were in S phase 10 hr after ara-C or aphidicolin removal, respectively (Additional file 1). By contrast, treatment with MTX resulted in 51% of the cells to be in S phase, while 28% were in G0-G1 phase, 10 hr after drug removal (Figure 1A). The ratio of cells in S phase remained higher than that in G1 phase up to 30 hr following MTX removal. This combination of an increase of cells in S phase and a decrease of cells in G1 phase resulted in a wave of cells in G2-M between 10 and 24 hr after MTX removal. The synchronization of cells in S phase by MTX was reversible as the pattern of cell cycle progression of MTX-treated cells was similar to that of untreated cells 48 hr after drug removal (Figure 1A). Our results thus suggest that MTX is more effective in synchronizing DHDK12 cells in S phase than ara-C or aphidicolin. Consequently, the efficacy of MTX in synchronizing cells in S phase was then tested in the HT29 cell line.

In HT29 cell line, the effect of MTX on cell cycle progression was slightly different. As illustrated in Figure 1B, cells began to accumulate in S phase almost immediately after MTX removal. While the rate of cells in S phase was 18% without treatment (Figure 1B), this rate reached 55% 6 hr after MTX removal and decreased thereafter to reach the ratio of untreated cells 24 hr after MTX removal.

Taken together, these observations indicate that the pattern of cell cycle synchronization after MTX removal is specific for each cell line. Because we hypothesize that gene transfer efficiency is improved by potent cell cycle synchronization, the time window for transduction experiments with the β-*gal *reporter gene should be different between the two cell lines.

### Improvement of gene transfer efficiency in synchronized cell

**Figure 2:**
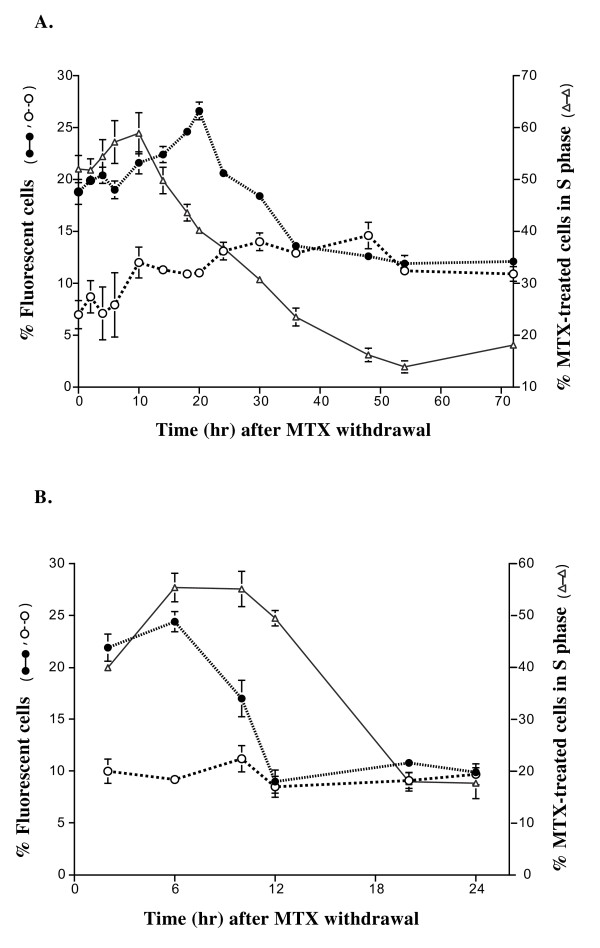
**Infection efficiency of the β-*gal *retroviral vector**. DHDK12 cells (**A**) and HT29 cells **(B**) were treated for 24 hr with (filled circle) or whithout (open circle) MTX. Cells were transduced with TG 5391 at the indicated times after MTX removal. The level of β-galactosidase activity was obtained 48 hr after the transduction by flow cytometry analysis using FDG, a fluorescent substrate of β-galactosidase. The percentage of cells in S phase (open triangle) at various time after MTX removal was determined by flow cytometry analysis of DNA content. Data are expressed as the mean ± SE from at least three separate experiments.

To determinate the optimal period for gene transfer in synchronized cells, we used the β-*gal *reporter gene. The rate of DHDK12 cells transduced with the β-*gal *gene was 3% with X-Gal staining while it was 10% with FDG in flow cytometry (data not shown). The treatment of DHDK12 cells with MTX improved retroviral gene transfer efficiency. Figure 2 shows that the level of transduction increased in cells synchronized in S phase. The highest level of transduction was obtained in the cells infected 20 hr after MTX removal. At that time, the proportion of transduced cells was 26% for cells treated with MTX, while it was 11% in untreated cells (Figure 2A). In the MTX-treated cell population, 44% of cells were in S phase. When the cell cycle distribution of MTX-treated cells returned to the control value 54 hr after drug removal, the efficiency of transduction became similar to that of control cells (Figure 2A). Thus, the optimal period to improve transduction efficiency of reporter gene in synchronized cells was obtained between 12 and 32 hr after drug removal.

Similar experiments were performed in HT29 cells. Accumulation of HT29 cells in S phase was observed almost immediately after drug washout. Accordingly, the highest transduction rate for β-*gal *gene was observed 6 hr after drug washout (Figure 2B). The efficiency of transduction was comparable to the control cells 12 hr after drug washout (Figure 2B).

**Figure 3:**
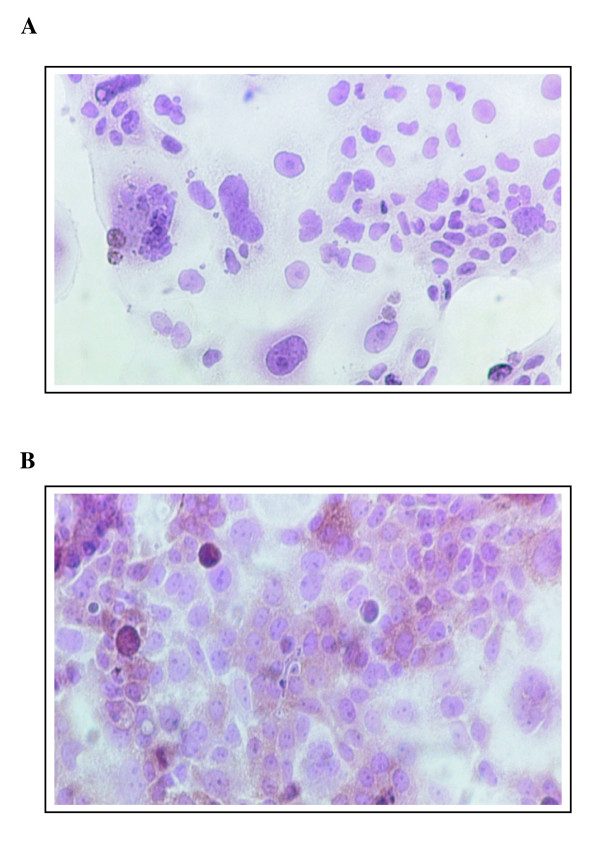
**Detection of HSV-TK protein**. DHDK12 cells (**A**) and DHDK12 cells transduced with the HSV-*tk *retroviral vector (**B**) were immunostained for HSV-TK. Cells seeded on chamber were transduced with TG 9344. After 48 hr, cells were fixed with 4% paraformaldehyde and stained with a mouse monoclonal 4C8 antibody against HSV-TK protein.

**Figure 4:**
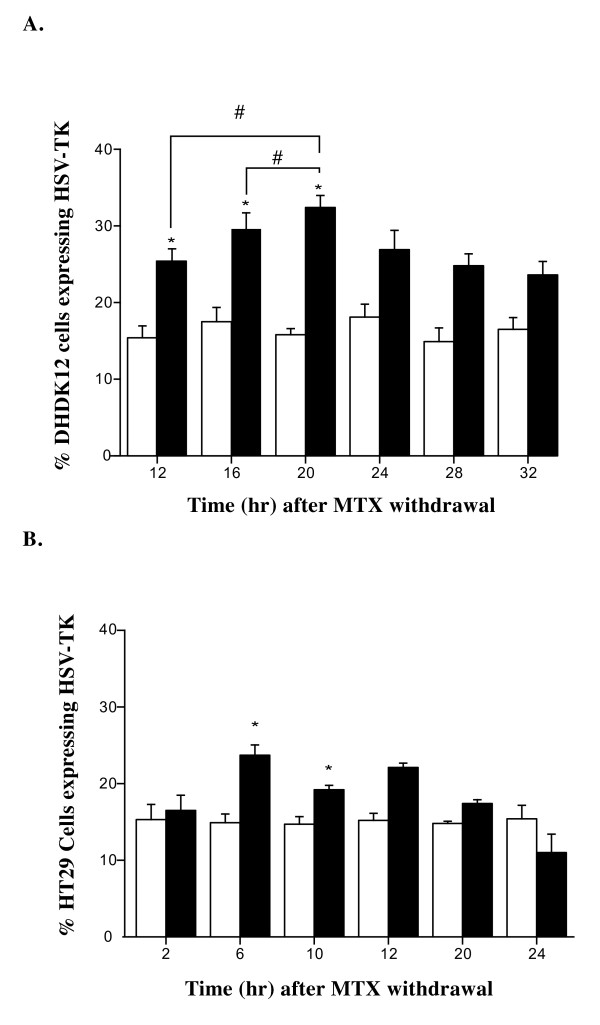
**Infection efficiency of the HSV-*tk *retroviral vector**. DHDK12 cells (**A**) and HT29 cells (**B**) were treated for 24 hr with (filled square) or without (open square) MTX. Cells were transduced with TG 9344 at the indicated times after MTX washout. The HSV-TK expression level was determined 48 hr after transduction by flow cytometry using a mouse monoclonal 4C8 antibody against HSV-TK protein. Data are expressed as the mean ± SE from at least three separate experiments. *P <.05 *vs*. untreated cells, # P <.05 *vs*. MTX-treated cells at 12 and 16 hr after MTX withdrawal.

As we first used the β-gal reporter gene to delineate the optimal period for subsequent HSV-tk gene transfer in synchronized cells, we focused our investigation for the transfer of the suicide gene HSV-*tk *in a time window for which the highest level of transduction with the β-gal reporter gene was obtained for each cell line. DHDK12 cells thus were treated with MTX and transduced with the HSV-*tk *gene from 12 to 32 hr after drug removal. Irrespective of the time used for transduction after MTX removal, the determination of the HSV-TK protein expression using flow cytometry or immunostaining was always performed 48 h after transduction to ensure protein expression of the transgene. As illustrated in Figure 3, immunostaining using peroxydase and DAB provided a brown intracellular precipitate in HSV-TK transduced cells. The rate of fluorescent untreated DHDK12 cells (control cells) expressing HSV-TK as measured by flow cytometry was 15% (Figure 4A). As observed for the β-*gal *reporter gene, the highest transduction rate in MTX-treated cells obtained after 20 hr of drug washout was 30% while it was 15% in control cells (Figure 4A).

For HT29 cells, transduction efficiency with HSV-TK was maximal at 6 hr after drug washout and reached 22% while it was 15% in untreated cells (Figure 4B). Therefore according to the host cell cycle, we found that pre-treatment with MTX resulted in improved gene transfer efficiency in these two cells lines.

### Enhancement of apoptosis in synchronized cell

**Figure 5:**
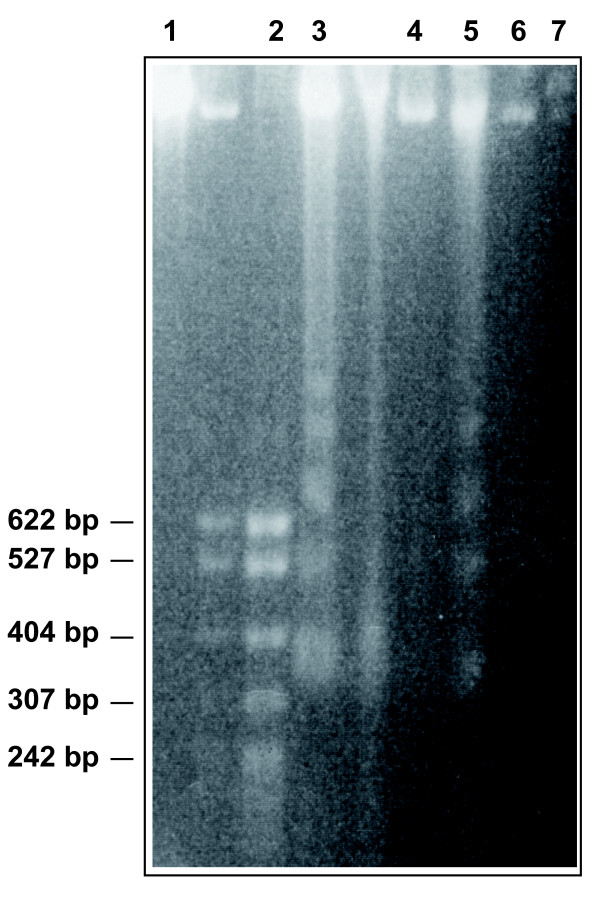
**Internucleosomal DNA fragmentation induced by GCV**. Lane 1 and lane 4 show DHDK12 cells and HT29 cells transduced with TG 9344 and treated for 96 hr with 20 μM GCV, respectively. Lane 3 and 5 show DHDK12 cells and HT29 cells transduced with TG 9344 after a 24 hr pretreatment with MTX and treated for 96 hr with 20 μM GCV, respectively. Lane 6 and 7 show DHDK12 cells and HT29 cells treated for 24 h with MTX, respectively. Lane 2 shows pBR 322 base pair size markers. Qualitative detection of DNA was achieved by ethidium bromide staining.

To determine whether the improvement of HSV-*tk *gene transfer efficiency by cell synchronization resulted into an increased GCV-mediated cell death, we measured the level of cell apoptosis after GCV treatment using annexin V-FITC. The presence of apoptosis observed with annexin V labeling was confirmed by the DNA fragmentation method (Figure 5). Annexin V labeling was increased in MTX-treated DHDK12 and HT29 cells transduced with HSV-*tk *gene and then treated for 72 hr by GCV.

**Figure 6:**
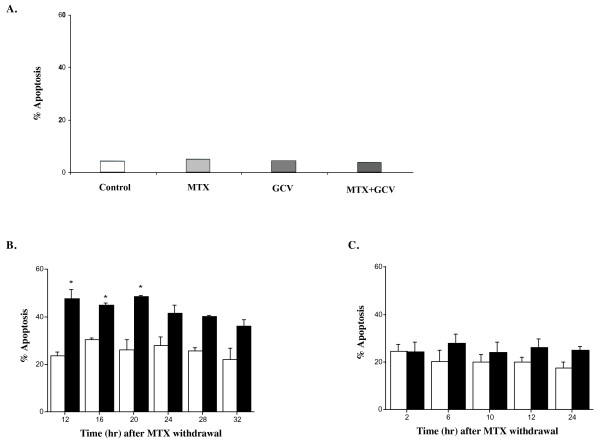
**Induction of apoptosis**. Untransduced DHDK12 cells (**A**) were treated with MTX, GCV or the combination of MTX plus GCV for 24 h. Transduced DHDK12 cells (**B**) and transduced HT29 cells (**C**) were treated for 24 hr with (filled square) or without (open square) MTX. Cells were transduced with TG 9344 at the indicated times after MTX washout and 48 hr after transduction were treated with 20 μM GCV for 72 hr. Quantitative detection of apoptosis was determined by biparametric flow cytometry analysis of fluorescein labeled-annexin V cells and PI. Apoptotic cells were annexin V positive, PI negative. Data are expressed as the mean ± SE from at least three separate experiments. * P <.05 *vs *untreated cells

In non-transduced cells, 5% of MTX treated cells were labeled for annexin V-FITC after treatment by GCV (Figure 6A). This corresponds to the intrinsic toxicity of MTX.

The percentage of MTX-treated DHDK12 cells undergoing apoptosis (Annexin V+, PI-) was two fold higher after MTX withdrawal (46% vs. 23% in the untreated cell population). The difference was maximal in cells transduced 20 hr after MTX withdrawal (Figure 6B).

In HT29 cells, the maximum percentage of MTX-treated cells undergoing apoptosis was 28% while it was 20% in untreated cells. The highest level of cell apoptosis was maximal 6 hr after MTX withdrawal (Figure 6C).

## Discussion

The objective of this work was to improve the efficiency of retroviral transfer of the suicide gene HSV-*tk *in colon cancer cells. This aim was achieved through the pharmacological control of the target cells cell cycle. Our results are consistent with previous reports showing that retroviral-mediated gene transfer depends on the cell cycle of target cells. The nuclear transfer of the pre-integrative viral complex is a strong limit to the efficiency of defective amphotrophic retroviral vectors derived from murine leukemia virus (MuLV). This step is possible only through the metaphasic breakdown of the nuclear membrane [14,16,30]. Therefore, the integration of retroviral DNA during cell division has only been evidenced when the doubling time of target cells was higher than the half-life of the virus [15]. As the half-life of MuLV-derived vectors is between 5.5 and 7.5 hr [31] and as the DHDK12 and HT29 cell lines have a doubling time of 28 hr [32] and 24 hr [33], respectively, our model meet this criterion. Our experimental design thus was adapted to study the efficiency of retroviral gene transfer after pharmacological control of the cell cycle.

Cell synchronization has been used to increase the number of cells accessible to drug targeting DNA and to improve the action of several anti-proliferative chemotherapies [20,23,24]. In this regard, experimental works have studied the synchronization in S phase of cancer cell lines by MTX, aphidicolin or ara-C. Aphidicolin and ara-C are reversible inhibitors of DNA polymerases [18,22]. MTX induces a reversible inhibition of dihydrofolate reductase, which is required for the *de novo *synthesis of nucleotides for DNA replication [34]. Our study showed a limited efficiency of ara-C or aphidicolin in DHDK12 cells. Moreover, a significant toxicity of aphidicolin, not compatible with an *in vivo *application, has been observed on several cancer cell lines [19,35]. We observed that non-toxic concentrations of MTX induced a reversible synchronization of DHDK12 and HT29 cells in early S phase (Figure 1). A 24 hr-treatment with MTX allowed increasing the rate of cells in S phase. The reversibility of MTX was confirmed as the cells returned to the normal cell cycle according to there doubling time. These results were in accordance to those obtained in others cell lines [36].

The reverse transcription of retroviral DNA can occur in several phases of the cell cycle [16]. However, the cells should be stimulated to divide before infection for efficient gene transfer [37]. According to the intracellular half-life of retroviral intermediates, the position of target cells relative to mitosis and the duration of S phase at the time of exposure both are critical to determine the efficiency of infection [38]. This assumption was supported by the difference in retroviral gene transfer improvement between DHDK12 and HT29 cell lines after cell synchronization by MTX. These two colon cancer cell lines exhibit a different pattern of cell cycle distribution after synchronization (Figure 1). We have observed that in HT29 cells the level of transgene expression, which was lower than that observed in DHDK12 cells, was strictly related to the peak of cells in S phase (Figure 2B). In DHDK12 cell line, the peak of cells in S phase was located 10 hr after the recovery and the infection efficiency was improved by 2-fold 20 hr after MTX removal (Figure 2A). The time difference between the maximum level of DHDK12 cells in S phase and the maximum efficiency of transduction could be related to the reverse transcription and integration of the viral DNA. Thus, the period of internalization and reverse transcription, which lasts 4 to 8 hours [16], must correspond to the interval necessary for cells synchronized in S phase to reach the G2-M phase to obtain the optimal integration of viral DNA. Our results indicate that the pattern of synchronization in DHDK12 cells at 20 hr after MTX removal is adapted to these criteria. In contrast to DHDK12 cells, HT29 cells synchronized in S phase reach more rapidly the G2-M phase, which may prevent optimal internalization and reverse transcription of the viral DNA in HT29 cells. This hypothesis is consistent with a model analyzing the kinetic of short half-life retrovirus mediated gene transfer [17]. Taken together, this allows delineating an optimal period for the retroviral gene transfer in synchronized target cells.

Quantitative detection of GCV-induced apoptosis was used to determine whether the increased efficiency of the HSV-*tk *retroviral gene transfer resulted in an increase in GCV-mediated cell death. The transduction rate of HSV-*tk *gene reached 30% in the DHDK12 cell line 20 hr after MTX removal, doubling the efficiency of retroviral gene transfer observed in untreated cells. Although the transduction rates of the β-gal reporter gene or the HSV-*tk *gene may appear rather low, they constitute a two-fold increase compared with the transduction rates previously described [12,13]. Indeed, in the aforementioned studies, the fraction of infected cells was less than 10% whereas in our experimental design it reached 30% in the DHDK12 cell line 20 hr after MTX removal. Because Chen *et al. *[9] have previously demonstrated that a higher level of HSV-TK expression correlates with greater bystander effect leading to increased cell killing, the increased transduction rate that we reached in our study could enhance GCV-mediated cell death. Consistently, our results show that the number of cells in apoptosis was higher than the number of cells expressing HSV-TK indicating greater bystander effect. Altogether, these observations indicate that improvement of transduction efficiency may represent a key step in retroviral suicide gene therapy by increasing both suicide gene expression and bystander effect.

We acknowledge nevertheless that this study has some limitations. Indeed, MTX was less efficient in HT29 cells than in DHDK12 cells in improving retroviral gene transfer and subsequently cell apoptosis after GCV treatment. This could be explained by an adverse effect of MTX metabolization leading to the inhibition of retroviral cycle. Indeed, the MTX metabolites have been shown to inhibit retroviral infection [39]. However, the rate of HT29 transduced cells undergoing apoptosis after GCV treatment increased from 20% to 28% in cells pre-treated with MTX. We are currently investigating whether a rescue strategy could antagonize the inhibitory effect of MTX metabolites on retroviral infection.

## Conclusions

We show here that cell synchronization may improve the efficacy of retroviral suicide gene transfer in a human and a murine colon cancer cell lines. Because the effect of cell synchronization on retroviral gene transfer differs between the two colon cancer cell lines used in this study, further investigations in more colon cancer cell lines are needed to draw definitive conclusion on the improvement of retroviral gene transfer after cell synchronization. Nevertheless, we demonstrate in the present study that this improvement increases the level of apoptosis induced with GCV treatment. This approach could be fruitful in colon cancer liver metastases because tumor cells are proliferating in a quiescent parenchyma. Therefore, we are currently assessing in a rat model of liver tumors whether this strategy could improve the antitumoral efficacy of cancer gene therapy using defective retroviral vectors.

## Competing interests

The author declares that they have no competing interests.

## Authors' contributions

LF performed the experiments and drafted the manuscript. AK, CP, SN and DG performed the experiments and participated in the interpretation of data. JL performed the experiments. CP, BN and JFE participated in the coordination of the study. RM conceived of the study, and participated in its design and coordination and drafted the manuscript. All authors read and approved the final manuscript.

## Supplementary Material

Additional file 1 **Ara-C and Aphidicolin mediated effects on DHDK12 cell cycle**. DHDK12 cells were treated with 0.075 μM ara-C or 25 μ M aphidicolin for 24 h. The percentage of cells in S phase (open square: aphidicolin; filled square: ara-C) and in G1 phase (open triangle: aphidicolin; filled triangle: ara-C) at various time after ara-C or aphidicolin removal was determined by flow cytometry analysis of DNA content Click here for file

## References

[B1] Edelstein ML Abedi MR Wixon J Gene therapy clinical trials worldwide to 2007--an update J Gene Med 2007 9 833 842 10.1002/jgm.1100 17721874

[B2] Thomas CE Ehrhardt A Kay MA Progress and problems with the use of viral vectors for gene therapy Nat Rev Genet 2003 4 346 358 10.1038/nrg1066 12728277

[B3] Sandmair AM Loimas S Puranen P Immonen A Kossila M Puranen M Hurskainen H Tyynela K Turunen M Vanninen R Lehtolainen P Paljarvi L Johansson R Vapalahti M Yla-Herttuala S Thymidine kinase gene therapy for human malignant glioma, using replication-deficient retroviruses or adenoviruses Hum Gene Ther 2000 11 2197 2205 10.1089/104303400750035726 11084677

[B4] Rainov NG A phase III clinical evaluation of herpes simplex virus type 1 thymidine kinase and ganciclovir gene therapy as an adjuvant to surgical resection and radiation in adults with previously untreated glioblastoma multiforme Hum Gene Ther 2000 11 2389 2401 10.1089/104303400750038499 11096443

[B5] Culver KW Ram Z Wallbridge S Ishii H Oldfield EH Blaese RM In vivo gene transfer with retroviral vector-producer cells for treatment of experimental brain tumors Science 1992 256 1550 1552 10.1126/science.1317968 1317968

[B6] Cheng YC Huang ES Lin JC Mar EC Pagano JS Dutschman GE Grill SP Unique spectrum of activity of 9-[(1,3-dihydroxy-2-propoxy)methyl]-guanine against herpesviruses in vitro and its mode of action against herpes simplex virus type 1 Proc Natl Acad Sci USA 1983 80 2767 2770 10.1073/pnas.80.9.2767 6302704 PMC393909

[B7] Garcia-Rodriguez L Abate-Daga D Rojas A Gonzalez JR Fillat C E-cadherin contributes to the bystander effect of TK/GCV suicide therapy and enhances its antitumoral activity in pancreatic cancer models Gene Ther 2011 18 73 81 10.1038/gt.2010.114 20720574

[B8] Mesnil M Yamasaki H Bystander effect in herpes simplex virus-thymidine kinase/ganciclovir cancer gene therapy: role of gap-junctional intercellular communication Cancer Res 2000 60 3989 3999 10945596

[B9] Chen CY Chang YN Ryan P Linscott M McGarrity GJ Chiang YL Effect of herpes simplex virus thymidine kinase expression levels on ganciclovir-mediated cytotoxicity and the "bystander effect" Hum Gene Ther 1995 6 1467 1476 10.1089/hum.1995.6.11-1467 8573619

[B10] Smiley WR Laubert B Howard BD Ibanez C Fong TC Summers WS Burrows FJ Establishment of parameters for optimal transduction efficiency and antitumor effects with purified high-titer HSV-TK retroviral vector in established solid tumors Hum Gene Ther 1997 8 965 977 10.1089/hum.1997.8.8-965 9195219

[B11] Terazaki Y Yano S Yuge K Nagano S Fukunaga M Guo ZS Komiya S Shirouzu K Kosai K An optimal therapeutic expression level is crucial for suicide gene therapy for hepatic metastatic cancer in mice Hepatology 2003 37 155 163 10.1053/jhep.2003.50018 12500200

[B12] Caruso M Panis Y Gagandeep S Houssin D Salzmann JL Klatzmann D Regression of established macroscopic liver metastases after in situ transduction of a suicide gene Proc Natl Acad Sci USA 1993 90 7024 7028 10.1073/pnas.90.15.7024 8346212 PMC47068

[B13] Kianmanesh AR Perrin H Panis Y Fabre M Nagy HJ Houssin D Klatzmann D A "distant" bystander effect of suicide gene therapy: regression of nontransduced tumors together with a distant transduced tumor Hum Gene Ther 1997 8 1807 1814 10.1089/hum.1997.8.15-1807 9358030

[B14] Hajihosseini M Iavachev L Price J Evidence that retroviruses integrate into post-replication host DNA Embo J 1993 12 4969 4974 8262039 10.1002/j.1460-2075.1993.tb06190.x PMC413757

[B15] Dolnikov A Wotherspoon S Millington M Symonds G Retrovirus vector production and transduction: modulation by the cell cycle J Gen Virol 2003 84 3131 3141 10.1099/vir.0.19099-0 14573819

[B16] Roe T Reynolds TC Yu G Brown PO Integration of murine leukemia virus DNA depends on mitosis Embo J 1993 12 2099 2108 8491198 10.1002/j.1460-2075.1993.tb05858.x PMC413431

[B17] Andreadis S Fuller AO Palsson BO Cell cycle dependence of retroviral transduction: An issue of overlapping time scales Biotechnol Bioeng 1998 58 272 281 10.1002/(SICI)1097-0290(19980420)58:2/3<272::AID-BIT23>3.0.CO;2-D 10191401

[B18] Springett GM Moen RC Anderson S Blaese RM Anderson WF Infection efficiency of T lymphocytes with amphotropic retroviral vectors is cell cycle dependent J Virol 1989 63 3865 3869 2788225 10.1128/jvi.63.9.3865-3869.1989 PMC250981

[B19] Sen S Erba E D'Incalci M Synchronisation of cancer cell lines of human origin using methotrexate Cytometry 1990 11 595 602 10.1002/cyto.990110506 2379450

[B20] Toffoli G Corona G Gigante M Boiocchi M Inhibition of Pgp activity and cell cycle-dependent chemosensitivity to doxorubicin in the multidrug-resistant LoVo human colon cancer cell line Eur J Cancer 1996 32A 1591 1597 8911123 10.1016/0959-8049(96)00113-x

[B21] Erba E Sen S Lorico A D'Incalci M Potentiation of etoposide cytotoxicity against a human ovarian cancer cell line by pretreatment with non-toxic concentrations of methotrexate or aphidicolin Eur J Cancer 1992 28 66 71 10.1016/0959-8049(92)90387-H 1314631

[B22] Chresta CM Hicks R Hartley JA Souhami RL Potentiation of etoposide-induced cytotoxicity and DNA damage in CCRF-CEM cells by pretreatment with non-cytotoxic concentrations of arabinosyl cytosine Cancer Chemother Pharmacol 1992 31 139 145 10.1007/BF00685101 1333370

[B23] Matranga CB Shapiro GI Selective sensitization of transformed cells to flavopiridol-induced apoptosis following recruitment to S-phase Cancer Res 2002 62 1707 1717 11912144

[B24] Yoshimura K Yamauchi T Maeda H Kawai T Cisplatin, vincristine, methotrexate, peplomycin, etoposide (COMPE) therapy for disseminated germ cell testicular tumors Int J Urol 1997 4 47 51 10.1111/j.1442-2042.1997.tb00139.x 9179666

[B25] De Godoy JL Malafosse R Fabre M Mitchell C Mehtali M Houssin D Soubrane O A preclinical model of hepatocyte gene transfer: the in vivo, in situ perfused rat liver Gene Ther 2000 7 1816 1823 10.1038/sj.gt.3301313 11110413

[B26] De Godoy JL Malafosse R Fabre M Mehtali M Houssin D Soubrane O In vivo hepatocyte retrovirus-mediated gene transfer through the rat biliary tract Hum Gene Ther 1999 10 249 257 10.1089/10430349950019039 10022549

[B27] Gray JW Coffino P Cell cycle analysis by flow cytometry Methods Enzymol 1979 58 233 248 423764 10.1016/s0076-6879(79)58140-3

[B28] Saalmuller A Mettenleiter TC Rapid identification and quantitation of cells infected by recombinant herpesvirus (pseudorabies virus) using a fluorescence-based beta-galactosidase assay and flow cytometry J Virol Methods 1993 44 99 108 10.1016/0166-0934(93)90012-G 8227283

[B29] Wei SJ Chao Y Hung YM Lin WC Yang DM Shih YL Ch'ang LY Whang-Peng J Yang WK S- and G2-phase cell cycle arrests and apoptosis induced by ganciclovir in murine melanoma cells transduced with herpes simplex virus thymidine kinase Exp Cell Res 1998 241 66 75 10.1006/excr.1998.4005 9633514

[B30] Coffin JM Retrovirus restriction revealed Nature 1996 382 762 763 10.1038/382762a0 8752269

[B31] Andreadis ST Brott D Fuller AO Palsson BO Moloney murine leukemia virus-derived retroviral vectors decay intracellularly with a half-life in the range of 5.5 to 7.5 hours J Virol 1997 71 7541 7548 9311834 10.1128/jvi.71.10.7541-7548.1997 PMC192101

[B32] Dunnington DJ Buscarino C Gennaro D Greig R Poste G Characterization of an animal model of metastatic colon carcinoma Int J Cancer 1987 39 248 254 10.1002/ijc.2910390221 3804494 PMC7165865

[B33] Forgue-Lafitte ME Coudray AM Breant B Mester J Proliferation of the human colon carcinoma cell line HT29: autocrine growth and deregulated expression of the c-myc oncogene Cancer Res 1989 49 6566 6571 2684395

[B34] Abonyi M Prajda N Hata Y Nakamura H Weber G Methotrexate decreases thymidine kinase activity Biochem Biophys Res Commun 1992 187 522 528 10.1016/S0006-291X(05)81525-6 1520343

[B35] D'Anna JA Crissman HA Jackson PJ Tobey R Time-dependent changes in H1 content, H1 turnover, DNA elongation, and the survival of cells blocked in early S phase by hydroxyurea, aphidicolin, or 5-fluorodeoxyuridine Biochemistry 1985 24 5020 5026 10.1021/bi00340a010 2934087

[B36] D'Incalci M Erba E Sen S Rabbone ML Perlangeli MV Masera G Conter V Induction of partial synchronization of leukemia cells by continuous infusion of low-dose methotrexate followed by citrovorum factor J Natl Cancer Inst 1989 81 1509 1510 10.1093/jnci/81.19.1509 2674461

[B37] Miller DG Adam MA Miller AD Gene transfer by retrovirus vectors occurs only in cells that are actively replicating at the time of infection Mol Cell Biol 1990 10 4239 4242 2370865 10.1128/mcb.10.8.4239-4242.1990 PMC360961

[B38] Andreadis ST Palsson BO Kinetics of retrovirus mediated gene transfer: the importance of intracellular half-life of retroviruses J Theor Biol 1996 182 1 20 10.1006/jtbi.1996.0140 8917734

[B39] Balk SD Mitchell RS LeStourgeon D Hoon BS Thymidine and hypoxanthine requirements for the proliferation of normal and Rous sarcoma virus-infected chicken fibroblasts in the presence of methotrexate Cancer Res 1979 39 1854 1856 218735

