# Supplementary figures and images for: Improved retroviral suicide gene transfer in colon cancer cell lines after cell synchronization with methotrexate
Source: J Exp Clin Cancer Res. 2011 Oct 4;30(1):92. doi: 10.1186/1756-9966-30-92 (PMC3199255; doi:10.1186/1756-9966-30-92)

% of cells in phase of the cell cycle

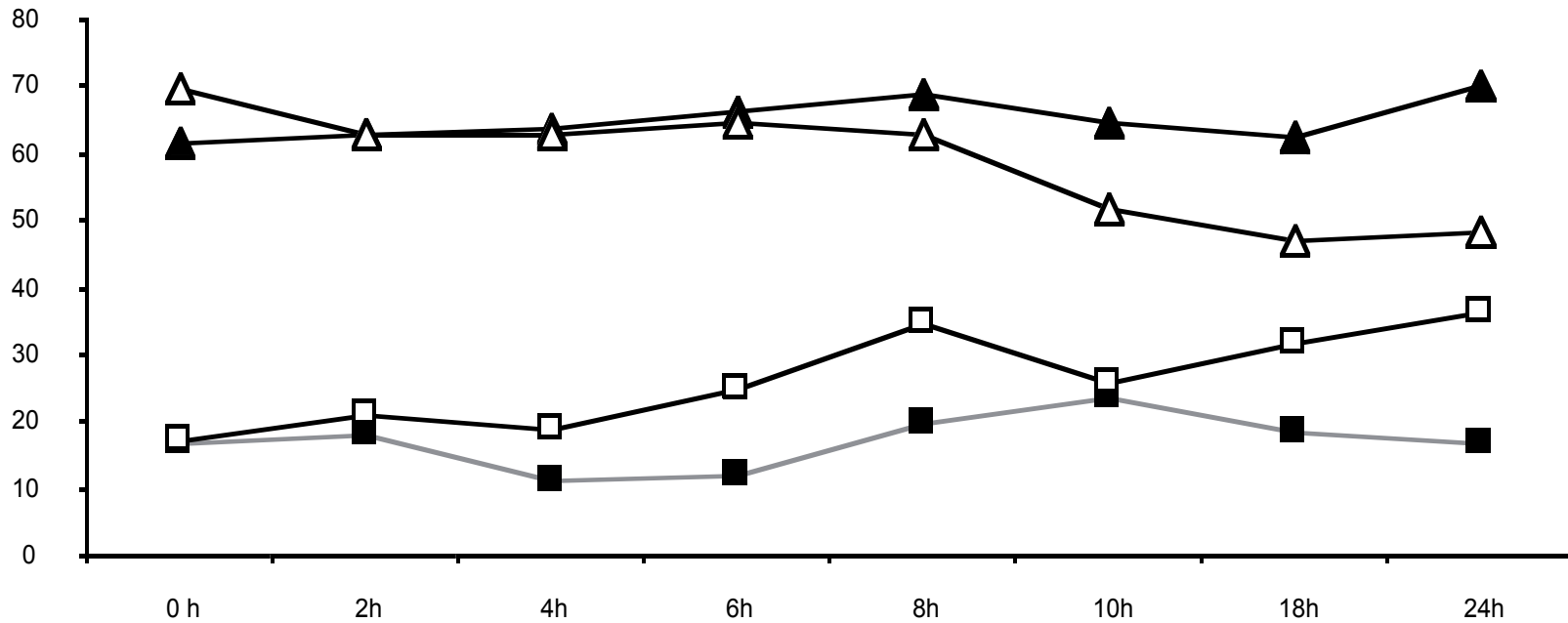

Time (hr) after drug withdrawal

Supplement: Additional file 1 — Ara-C and Aphidicolin mediated effects on DHDK12 cell cycle. DHDK12 cells were treated with 0.075 μM ara-C or 25 μ M aphidicolin for 24 h. The percentage of cells in S phase (open square: aphidicolin; filled square: ara-C) and in G1 phase (open triangle: aphidicolin; filled triangle: ara-C) at various time after ara-C or aphidicolin removal was determined by flow cytometry analysis of DNA content [file 1756-9966-30-92-S1.PDF]
